# Phenoplasticity of Essential Oils from Two Species of *Piper* (Piperaceae): Comparing Wild Specimens and Bi-Generational Monoclonal Cultivars

**DOI:** 10.3390/plants11131771

**Published:** 2022-07-04

**Authors:** Ygor Jessé Ramos, Jéssica Sales Felisberto, João Gabriel Gouvêa-Silva, Ulisses Carvalho de Souza, Claudete da Costa-Oliveira, George Azevedo de Queiroz, Elsie Franklin Guimarães, Nicholas John Sadgrove, Davyson de Lima Moreira

**Affiliations:** 1 Natural Products and Biochemistry Laboratory, Botanical Garden of Rio de Janeiro Research Institute, Rio de Janeiro Botanical Garden, Rio de Janeiro 22460-030, Brazil; ygorjesse@jbrj.gov.br (Y.J.R.); jessicka.salles@gmail.com (J.S.F.); joaogabrielfarma@outlook.com (J.G.G.-S.); usouza@jbrj.gov.br (U.C.d.S.); 2 Institute of Biology, State University of Rio de Janeiro, Rio de Janeiro 20550-013, Brazil; deteoliveira@hotmail.com (C.d.C.-O.); georgeazevedo08@gmail.com (G.A.d.Q.); elsie.guimaraes@jbrj.gov.br (E.F.G.); 3 Jodrell Science Laboratory, Royal Botanic Gardens Kew, Richmond TW9 3DS, UK

**Keywords:** aromatic plant, Piperaceae, terpenes, chemodiversity, chemical plasticity, phenoplasticity

## Abstract

This study tested the hypothesis that “clonal chemical heritability is a crucial factor for the conservation of chemical uniformity of *Piper* essential oils in controlled monoclonal cultivation”. We asexually propagated first and second-generation clones of two medicinal and aromatic species, *Piper gaudichaudianum* Kunth and *Piper mollicomum* Kunth (Piperaceae), for use as experimental models since they show high chemical plasticity in the wild. Leaves from wild specimens of both species, and their respective cultivated specimens, were hydrodistilled in a Clevenger-type apparatus to produce essential oils (EOs). EOs were chemically characterised by GC-MS and GC-FID. The analysis identified 63 compounds in EO of *P. mollicomum*, which were predominantly monoterpenes, and 59 in EO of *P. gaudichaudianum*, which were predominantly sesquiterpenes. Evaluation of chemical diversity and oxi-reduction indices showed a loss of chemical homology across the intergenerational cline. Chemometric analysis indicated higher chemical plasticity between wild and intergenerational specimens of *P. mollicomum*, than for *P. gaudichaudianum*. EO compounds were significantly less oxidized throughout the generations in both species. Therefore, while clonal heritability is crucial to chemical homology, significant chemical plasticity is likely to occur when cultivated from wild specimens.

## 1. Introduction

In Brazil, species in the genus *Piper* are among the most versatile of the useful plants in the country [1]. For example, the two species *Piper mollicomum* Kunth and *P. gaudichaudianum* Kunth have extensive ritualistic and medicinal uses. They are known synonymously by the vernacular name “Jaborandi” by local herbalists [2,3,4]. It is not uncommon for the same name to be used for two or more taxa with similar appearances that are used interchangeably for the same end uses. This is corroborated by ethnobotanical studies of Jaborandi that highlighted overlap of use, in particular, in the initiation of people into religions of Brazilian African origin. Furthermore, both species are used as ingredients for the bath of “amaci”, for aromatic drinks, or as an incense [5,6].

These two species of *Piper* have numerous therapeutic applications that are recognized in contemporary practice, as well as in history. Records made in 1888 describe the use of infructescences (the fruit clusters) from *P. mollicomum* for the treatment of diseases of the gastrointestinal tract, as well as for venereal diseases [7]. Modern records describe the use of its leaves in the treatment of liver diseases, for the relief of spine pain, in the reduction of intense menstrual flow [8,9,10,11,12], as well as antifungal [13], antibacterial [10,14], and antinociceptive [15] applications. Another organ that is used is the roots, which are extracted and applied as a local anesthetic for toothache in the form of an aqueous infusion [7,9,11,16]. The other species, *P. gaudichaudianum*, is similarly an anti-inflammatory agent, also used in the relief of toothache and in the treatment of liver diseases [17]. The pharmacological potential of the extract of this species is also corroborated by in vitro studies that demonstrate fungicidal, larvicidal, anti-inflammatory and analgesic activities [18,19,20].

Regarding essential oils (EOs), those obtained from the leaves of *P. gaudichaudianum* have shown in vitro larvicidal, insecticidal anti-inflammatory and cytotoxic activities [21]. There is also evidence of moderate antibacterial activity of EOs produced from the leaves of *P. mollicomum* [16]. Previous chemical studies show that the EOs from leaves of *P. mollicomum* and *P. gaudichaudianum* are predominantly terpenoid with traces of arylpropanoids, however their chemical constitutions vary according to their collection site, season, and time of day [15,21,22,23,24,25].

Most of the medicinal plants in Brazil that are highly regarded have not yet been put into cultivation [26]. Consequently, the increasing use of medicinal plants that are wild harvested is putting the native populations under considerable pressure, and without intervention they may become threatened. This is already a significant problem globally, considering that 40% of the world’s flora is at risk of extinction and/or genetic erosion, due to excessive biota harvesting [27]. In anticipation that such problems may affect the medicinal species *P. mollicomum* and *P. gaudichaudianum*, we initiated studies related to agronomic practices.

The cultivation of medicinal plants outside their niche habitat is unregulated in Brazil, but there are efforts to acquire scientific agronomic knowledge in small-scale cultivations that remain close to natural growing land areas. Indeed, there are a few cultivation approaches with medicinal species from the genus *Piper* [28,29,30]. However, the circulation of seedlings and seeds of medicinal plants around Brazil, either at the community level or regionally, antagonizes the understanding and standardization of chemical and biological phenotypes within and across species [31,32]. For example, it is well known that plants grown in non-natural environments can present qualitative and quantitative variations in the production of specialized metabolites, because the chemical phenotypes are not just interconnected with chemical heritability [33], since extrinsic factors are also significant.

In this regard, the concept of “chemical heritability” is used to define the ratio of chemical diversity over genetic diversity within a population [34,35]. Chemical heritability recognizes the influence of exogenous factors in bringing about chemical diversity within a population. Through different concepts and in practice, chemical heritability in plants can be further evaluated from sexual (seed) and asexual (clonal) propagation. In the first, genetic factors may still influence the chemical diversity that is measured from cultivated plants. However, in the second method of clonal propagation, the elimination of genetic diversity across replicates is the key to understanding the phenomena of chemical phenotypic plasticity, and obviously, the mechanisms of chemodiversity [36,37]. Thus, clonal propagation is used in the current study as the chosen method to answer the question: “Is clonal chemical heritability a crucial factor for the conservation of chemical and chemodiversity characteristics of *Piper* foliar essential oils in controlled cultivation?” To answer this question, this work aims to evaluate the clonal heritability of EO chemodiversity between wild and intergenerational specimens *P. gaudichaudianum* and *P. mollicomum*.

## 2. Results

The EO composition and yield obtained from the leaves of *P. gaudichaudianum* wild (PGW), first (PGF) and second (PGS) generations, as well as *P. mollicomum* wild (PMW), first (PMF) and second (PMS) generations are shown in the Table 1. The EOs were slightly yellow in colour. The lowest yield was registered for PMF (0.12%), and the highest for PMW (0.86%). The yields of *P. gaudichaudianum* ranged from 0.13–0.18%.

### 2.1. Chemical Variation in P. gaudichaudianum

EOs obtained from the leaves of PGW contained 59 identifiable compounds, corresponding to 98.01% according to the quantitative method used. Among these, 97.54% were identified as sesquiterpenes, while monoterpenes were less than 1%. The main identified constituents were bicyclogermacrene (14.23–28.16%), Z-eudesma-6,11-diene (2.31–4.32%), α-terpineol (4.87–5.62%), *E*-caryophyllene (2.34–8.43%), and the oxygenated sesquiterpenes E-nerolidol (6.32–12.11%), viridiflorol (5.21–7.89%) and α-cadinol (3.21–9.32%) (Table 1).

**Figure 1:**
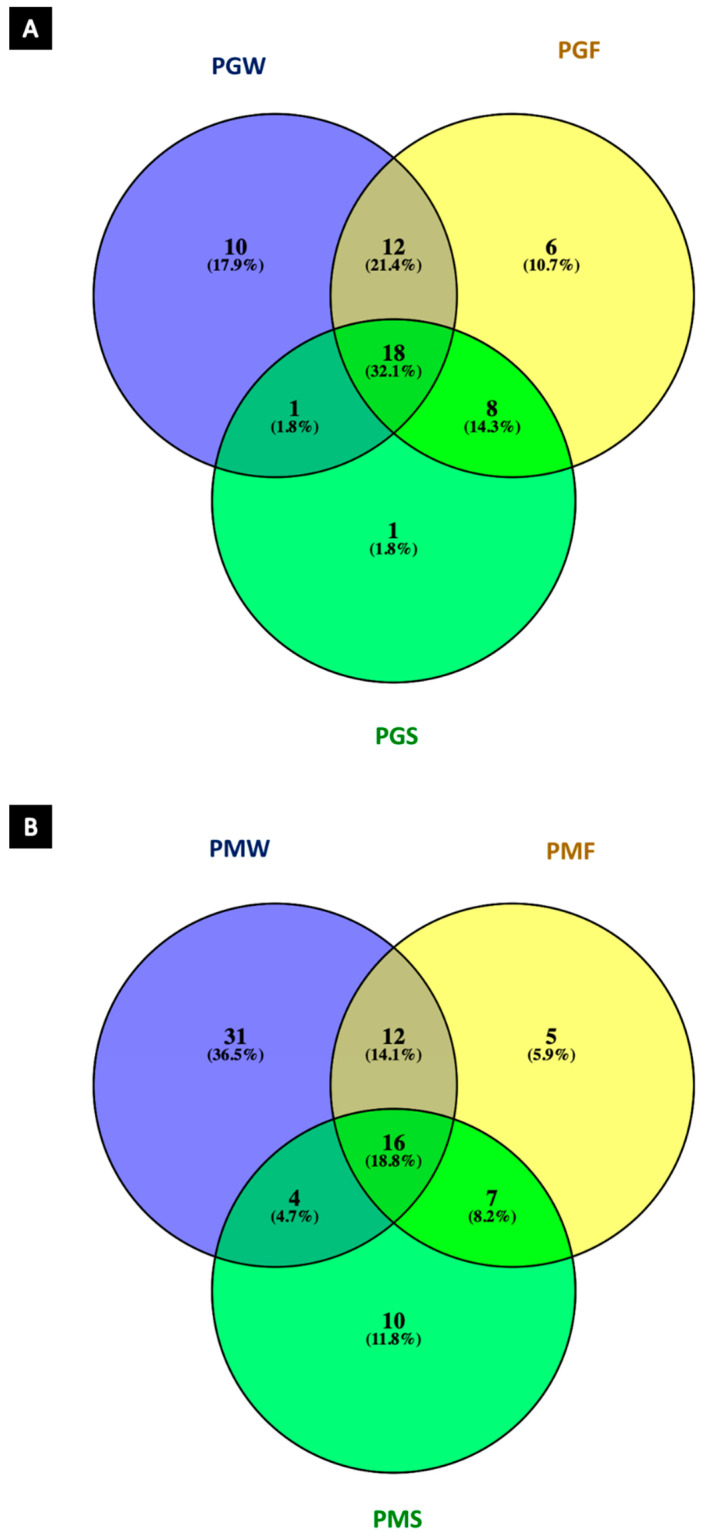
Venn diagram achieved from the constituents of the essential oil in the leaves of (**A**) *Piper gaudichaudianum* wild (PGW), first (PGF) and second (PGS) generations and (**B**) *Piper mollicomum* wild (PMW), first (PMF) and second (PMS) generations.

A qualitative analysis of the EOs of both species is presented in the form of a Venn diagram (Figure 1). The amount of overlap between chemical profiles across the three generations of the species is captured by this analysis. The profile of EOs from leaves of *P. gaudichaudianum* (Figure 1A) demonstrates that there are 18 compounds (32.10%) common to the three generations studied. However, at the level of individual specimens, these 18 compounds represent 67.36%, 67.86% and 81.33% of the total number of compounds in the profile of PGW, PGF and PGS, respectively. Six compounds for PGF were unique to this generation: α-pinene, α-ylangene, amorpha-4,7(11)-diene, β-calacorene, 5-*epi*-7-*epi*-α-eudesmol, and 7-epi-α-eudesmol. However, they are only minor compounds (<1%). For PGS, only one compound was exclusive to this sample, again in low relative percentage (*E*-β-farnesene, 1.73%). In contrast, the 30 major compounds that account for more than 80% of the total profile, are shared between PGW and PGF. This commonality is not seen in the final propagated clone (PGS), which only shares eight compounds with its progenitor (PGF).

### 2.2. Chemical Variation in P. mollicomum

A total of 63 compounds were successfully identified in the EOs of wild *P. mollicomum* (PMW), corresponding to 98.34% of the whole mass. EOs are dominated by monoterpenes (oxygenated and non-oxygenated; 79.40%), with a fraction of sesquiterpenes (oxygenated and non-oxygenated; 18.60%). Over the generations in cultivation, the class of identified compounds gradually became more sesquiterpenoid (first generation (PMF): 25.91%/second generation (PMS): 45.25%), with evident reciprocal declining of the monoterpene composition (PMF: 56.07/PMS: 37.33%). In addition, the diversity of the identified compounds in cultivated specimens was considerably reduced, particularly from PMF to PMS.

The chemical constitution of *P. mollicomum* EOs changed considerably across the clonal generations. The major compounds identified in the wild specimens (PMW) are the oxygenated monoterpenes 1,8-cineole (34.10%) and linalool (7.26%); and the non-oxygenated compounds, α-pinene (15.20%) and β-pinene (12.10%), as well as the oxygenated sesquiterpene E-nerolidol (2.12%). Alternatively, the EO compounds from the first generation (PMF) are linalool (37.88%), *Z*-linalool oxide (18.95%), *E*-caryophyllene (2.44%) and E-nerolidol (2.14%). 1,8-Cineole, α-pinene and β-pinene were detected, but at a low relative percentage compared to the wild samples. As a progression, the EOs from the second generation (PMS) are similar but with a higher amount of linalool (36.99%), E-nerolidol (11.39%) and benzyl benzoate (5.69%).

The Venn diagram for *P. mollicomum* (Figure 1B) shows 16 compounds that are common to all specimens (PMW and PMF/PMS; 18.80%), which represent 14.78%, 62.32% and 67.29% of the total content of EO, respectively. Five compounds were exclusive to PMF: *E*-linalool oxide, *E*-muurola-3,5-diene, *Z*-bisabolol-11-ol, *Z*-cadin-4-en-7-ol and eudesm-7(11)-en-4-ol. These exclusive compounds accounted for a small portion of the whole, with only E-linalool at >1%, in contrast with its *Z*- isomer. In the second generation (PMS) ten compounds are exclusive: *Z*-calamenene, zonarene, guaiol, humulene epoxide II, 10-*epi*-γ-eudesmol, *E*-isolongifolanone, *E*-sesquilavandulol, *epi*-α-cadinol, benzyl butanoate, 2-tridecanone (9.58% of the total EO). Four of these exclusive compounds were quantified as above 1%. Lastly, 28 compounds are shared between PMW and PMF; and 20 between PMW and PMS. The number increases to 23 when correlation is carried out only with PMF and PMS.

### 2.3. Chemometric Analysis

**Figure 2:**
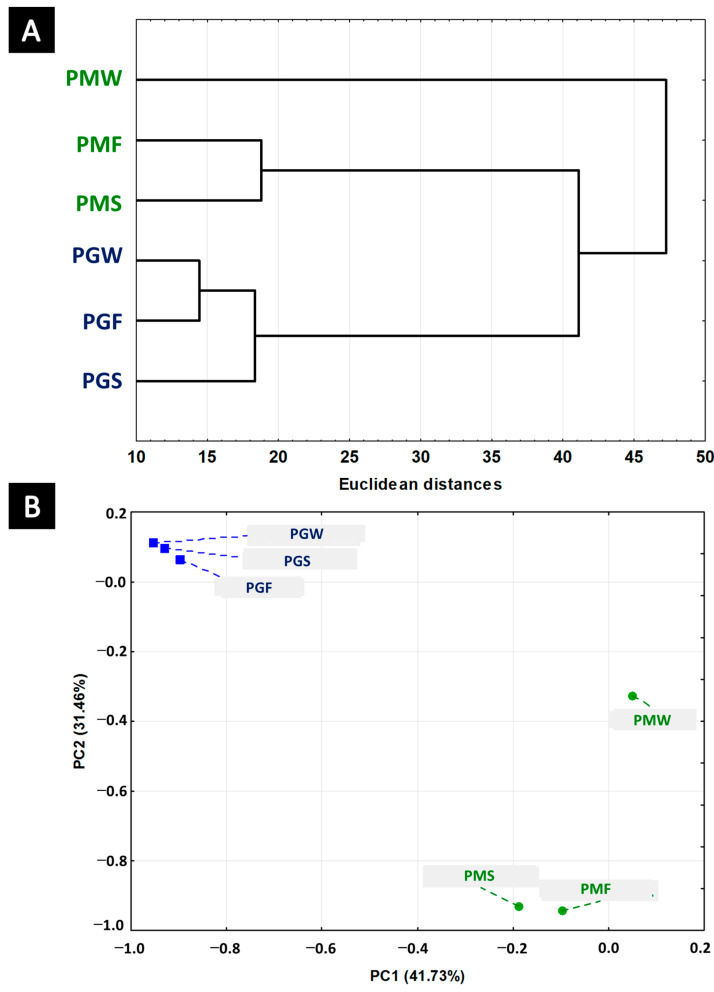
Dendrogram (**A**) and Biplot (PCA) (**B**) representing the similarity relationship of the essential oil compounds in the leaves of *Piper mollicomum* wild (PMW), first (PMF) and second (PMS) generations and *Piper gaudichaudianum* wild (PGW), first (PGF) and second (PGS) generations.

Figure 2 shows the results of the hierarchical cluster analysis (A) and the main component analysis (B) of chemical profiles from both the species and their intergenerational specimens. Figure 2 demonstrates that intergenerational differences between the clones was less of a discriminating factor, compared to interspecies differences. This clarifies that clonal heritability remains as a robust factor in chemical expression patterns. The first discriminating factors explained 74.18% (PC1: 43.72% + PC2: 31.46%) of the accumulated variation of the analysed data. The horizontal axes clearly separated the species by the chemical composition. For *P. gaudichaudianum* the most important chemical compound in the separation was bicyclogermacrene with negative charges in PC1 (−12.13) and positive in PC2 (1.28). For *P. mollicomum* it was linalool with negative charges in PC1 (−0.91) and PC2 (−12.84). The cyclic monoterpene 1,8-cineole, with positive charge on PC1 (0.99) and negative PC2 (−1.93), characterises the chemical plasticity showed by PMW.

### 2.4. Soil Characteristics

Table 2 demonstrates several inorganic characters of the native soils from which the wild specimens were collected. Compared to the propagation soils of the clones, the wild soils had a significantly lower pH, a lower salt content and a higher mineral content. The influence of these abiotic factors has not been examined in any further detail.

### 2.5. Chemodiversity and Micromolecular Analysis

The chemical data in Table 1 is evaluated using two ‘indices’, which is the Shannon index, and the recently developed oxy-reduction index (Ramos and Moreira Index: GM_RO_) [21]. These evaluations demonstrated a trend in the context of clonal chemical heritability.

The Shannon index changed across the generations, from wild to the second generation for both species. Specifically for *P. mollicomum* the difference is from 4.14 to 3.67 (*r*^2^ = 0.944) and for *P. gaudichaudianum* it went from 3.81 to 3.30 (*r*^2^ = 0.784). These results suggest a loss of chemodiversity from wild to cultivated (first and second generations). This loss of chemical diversity is more distinguished for *P. mollicomum* than *P. gaudichaudianum*.

The evaluation of the oxy-reduction characteristics of the two EO mixtures for *P. mollicomum* showed values from −3.73 to −2.55 (r^2^ = 0.9435) and for *P. gaudichaudianum* from −6.94 to −3.39. These results mean that EO mixtures are less oxidized from wild to cultivated (first and second generations); in other words, EO mixtures reduced with acclimatation. It is not clear if this acclimation was to the abiotic factors measured and summarized in Table 2, or if other factors are significant, such as water regime, light exposure, or others.

## 3. Discussion

The high sesquiterpene content of *P. gaudichaudianum* in the current study is in accordance with literature data for other species in Piperaceae [17,38]. Chemical studies of the same species are also in agreement, i.e., Peres et al., [39] analysed the EO from leaves of *P. gaudichaudianum* that was collected in Rio Grande do Sul (Brazil). They described *E*-nerolidol (22.4%) as the major constituent, and in minor relative percentages, *E*-caryophyllene (8.9%) and bicyclogermacrene (7.4%). That study also showed cytotoxic, genotoxic, and mutagenic effects that were correlated to the presence of *E*-nerolidol, α-humulene and *E*-caryophyllene [39].

Another paper on the same species and the same Brazilian State, demonstrated a very different chemical character of volatiles from *P. gaudichaudianum*. The profile was dominated by the arylpropanoid dillapiole and the sesquiterpene α-humulene [40]. That study was carried out over a year of sampling, registered less relative percentage of *E*-caryophyllene, however, compounds bicyclogermacrene and *Z*-eudesma−6,11-diene were not detected.

In other studies, similar chemotypic variation has been reported in *P. gaudichaudianum*. Dominant components vary across studies, such as longipinanol [41], δ-cadinene [42], 1-*epi*-cubenol [43], β-pinene [43,44], germacrene B [22,45,46], and viridiflorol [20]. These results reflect a high interspecies chemical plasticity.

A study managed by our group [21] showed nine chemotypes for *P. gaudichaudianum* (i.e., δ-cadinene, 1-*epi*-cubenol, longipinanol, viridiflorol, α-humulene, *E*-caryophyllene, germacrene B, dillapiole and bicyclogermacrene), confirming this pronounced phenotypic chemical plasticity. Furthermore, we showed that EOs from *P. gaudichaudianum* from Rio de Janeiro are normally the bicyclogermacrene chemotype. In the current work we have demonstrated that the bicyclogermacrene chemotype is conserved between first and second generations from a wild sample, confirming that the phenotypic expression for the group located in the city of Rio de Janeiro is likely a genotypic characteristic.

In relation to *P. mollicomum*, linalool is the major compound of the EO in this study, having been reported in all studied samples (7.26–36.99%). This monoterpene is commonly found in some species of the genus *Piper*, i.e., *P. aduncum* [47]; *P. jacquemontianum*, *P. multiplinervium*, and *P. darienense* [48]. Linalool is well known for its biological activities, as reported in the literature, such as antifungal activity against *Candida albicans* [49], antibacterial activity against *Staphylococcus aureus* and *Escherichia coli* [50], its action against periodontopathic and cariogenic bacteria [51], and its trypanocidal activity against trypomastigote forms of *Trypanosoma cruzi* [52]. Thus, it is suggested that the cultivation of *P. mollicomum* may provide significant amounts of a native lavender EO for market interest. The essential oil can be used as a key fragrance in the development of new aesthetic products.

Linalool also has an interesting ecological backstory. Literature reports showed that grape species exposed to high levels of UV light demonstrate an increase in the biosynthesis of this monoterpene. It is possible that UV light interacts with some enzymes, such as linalool synthase, which is involved in the bioconstruction process of cyclic and acyclic monoterpenes [53]. In contrast, other studies demonstrated that linalool is decreased when the plant is exposed to high UV radiation, while other cyclic monoterpenes such as camphor and 1,8-cineole are preserved [54]. UV radiation is not the only factor that is implicated in chemical expression. For example, Blande and Glinwood [55] identified an increase in linalool content in the vegetative parts of plants exposed to stress, due to abiotic factors.

Another compound of interest that is present in the chemical profile of EO from *P. mollicomum* is benzyl benzoate. This compound is rare in EO; however, it has already been described for species in the genus *Piper*, i.e., in EO produced from *P. retrofractum* (14.40%) and *P. sarmentosum* (49.50%) [56].

Other studies on the EO of *P. mollicomum* describe a volatile mixture that differs from our results. Santos et al., [38] published an EO composition (leaves collected in the city of Paraty, Rio de Janeiro State, Brazil) richer in oxygenated sesquiterpenes (39.04%) in higher content than non-oxygenated (21.29%). However, our group [24] described similar results on the composition of the *P. mollicomum* EO collected in Rio de Janeiro city. We found 1,8-cineole and linalool as the main compounds of the volatile mixture. Thus, it is suggested that there are multiple chemotypes for the species, even within the State of Rio de Janeiro.

Qualitative statistical analysis (*Venn* diagram) serves as a tool to ensure the chemical uniformity or inconsistency of EOs, as the influence of minor components can be more than is expected, such as by conferring synergistic effects [57,58,59]. Thus, chemotypes and phenotypic plasticity are important considerations in the context of commercialization of essential oils with associated health claims [60,61,62,63].

The high phenotypic plasticity of *P. mollicomum* has been studied in wild samples [24], but this is the first study of plants in a controlled environment. This finding contributes mainly to the medicinal and ritualistic use of *P. mollicomum* in Afro-Brazilian religious communities that cultivate samples around their sacred spaces. The matrices are, generally, obtained from both wild and propagated specimens, as we did in this study. Therefore, with consideration to loss or change in chemical composition, the expected therapeutic effects may alter [5,6].

Total compounds in *P. mollicomum* samples varied significantly, whereas in *P. gaudichaudianum* samples it is quite constant. Current evidence from our group shows that different *Piper* species present different chemical plastic responses at the same collection site [21,24,26]. We also have registered that *P. mollicomum* showed a greater acclimatization response to abiotic factors from day-to-day [26] and *P. gaudichaudianum* throughout the hours of the day [21].

The PCA analysis also showed *E*-nerolidol as a chemical biomarker for the species, with negative charges PC1 (−5.74) and PC2 (−1.53). *E*-Nerolidol is found in the specimens in great amounts (1.94–12.11%). Studies on EO with a high content of *E*-nerolidol can be found in species of *Piper* from the Brazilian Atlantic Forest. For example, *P. aduncum* L. (80.6–82.5%) [52,64], *P. claussenianum* (Miq.) C. DC. (81.4–83.3%) [65], and *P. gaudichaudianum* Kunth (22.10–22.40%) [25,39]. Chan et al., [66] report that *E*-nerolidol is widely used in the industry as an important product in the manufacture of cosmetics, foods, and pharmaceuticals. For species of the genus *Piper* rich in *E*-nerolidol, several biological activities have already been described, such as antileishmanial (promastigotes of *Leishmania amazonensis*, IC_50_ = 30.24 µg/mL) and antifungal (*Candida albicans*, MIC 0.20–1.26%) activity for *P. claussenianum* [65,67,68]. Then there is cytotoxic (Chinese hamster lung cells V79, IC_50_ = 4.0 µg/mL) [39] and larvicidal action against *Aedes aegypti* for *P. gaudichaudianum* [69].

In summary, it was possible to determine that the diversity of compounds decreased in the first and second generations, for both species. This fact may be related to the cultivation environment, since outside of its natural habitat these plants may be under stress promoted by predation, asides from the different soil composition and micro-climate. These results may suggest that ecological interactions in the natural habitat are important for maintaining chemical diversity [57,58]. We emphasize that there are several factors that modify the chemical composition of EOs, being abiotic and biotic factors [24,70]. The age difference between the cultivated and wild specimens of the two studied species is a limiting factor and should be better investigated on a larger time scale. However, the different plastic responses registered mainly for *P. mollicomum* reflect the complexity of the biosynthetic pathways vs. biotic and abiotic factors that are involved. Our findings are new for these two species of *Piper* but have been reported before for other species. For example, *Satureja hortensis* L., popularly known as garden savoury, showed a composition rich in the monoterpene carvacrol in cultivated specimens, but it was rich in thymol in the wild specimens [71], which are two compounds from the same biosynthesis pathway. Our results demonstrated a similar type of difference in the EO composition from leaves of *P. mollicomum*, but it is a dichotomy of 1,8-cineole/linalool.

In addition, it is known that selection leads to micro-evolutionary changes in the composition and diversity of specialized metabolites, which may occur over only a few generations in time scales relevant to ecological interactions and forms of cultivation [72,73,74,75]. The passing of generations in commercial crops may be an important criterion for determining permanent phenotypic characteristics in species, especially in the process of determining and differentiating chemotypes and genotypes, respectively. Therefore, this report characterized these changes based on this phenomenon in the propagation of *P. gaudichaudianum* and *P. mollicomum*.

The main concerns of the current study are the selection of the explant/matrices, cultivation conditions, medium composition, culture age, genotype, temporal variations, number of subcultures, use of phytohormones, and regeneration methods. These are aspects significant for evaluating the stability and genetic and epigenetic variation of plants regenerated through propagation [76,77]. There are two ways in which epigenetic processes can contribute to microevolution in natural populations. First, if heritable epigenetic variation is translated into phenotypic variation and into adjustment differences between individuals, then epigenetic processes can provide a second system of heritable variation for natural selection to act upon, such as one based on genetic variation. On the other hand, epigenetic variation, in contrast with genetic variation, can be altered directly by abiotic factors and consequently provide an additional route for accelerating evolutionary change [73,74]. These variations were observed quantitatively with the loss of chemodiversity with the two studied species of *Piper*. However, in the language of molecular biology the term “epigenetic inheritance” is used for mitotic and meiotic inheritance of epigenetic modifications. This term is confused in classical genetics and evolutionary biology in which the term inheritance is usually restrictive to the description of transgenerational inheritance through meiosis [78,79].

The adequacy of an experimental design can be a significant tool for the selection of chemical characteristics for use in human health. These can be an alternative to study epigenetic evolution in action, in which it is guaranteed that the genotypes of the same plant are subjected to different environments, their descendants are raised in a common environment for several generations; after which phenotypic and epigenetic differences are quantified and statistically compared. If we find that the descendants of those lineages that have been submitted to different environments remain phenotypically different, and at the same time, they show a significant shift in patterns of DNA methylation, gene, or protein expression, despite being still identical at the DNA level, this is going to be the best evidence for rapid evolution based on epigenetics. This field brings interesting perspectives to studies on natural products [73,77,80,81,82,83]. To exemplify, associating this notion with human food consumption, a study evaluated the process of domestication of wheat, from three subspecies of *Triticum turgidum* L. (wild emmer, emmer, and durum wheat). The authors qualitatively investigated a mixture of 51 central metabolites. A reduction in unsaturated fatty acids was observed, while there was a decrease in amino acids characterized in secondary domestication (that of durum wheat) [73]. Loss of these two chemical markers was essential for improvement to the sensory characteristics of this species and in industrial application. Correlating with our results, chemical characteristics of the EO had less oxidized metabolites over the generations and loss of chemical diversity that reveal more about the acclimatization process [73,74]. We believe that these insights are initial postulates to decipher chemodiversity using *Piper* as a model, as it is a species of rapid propagation.

## 4. Materials and Methods

### 4.1. Plant Material

Fresh leaves (100 g) and twelve cuttings (standardized in 3 nodes) were collected from each adult individuals (n = 3) of *Piper gaudichaudianum* Kunth and *P. mollicomum* Kunth in regions of the Atlantic Forest of Tijuca National Park, in the city of Rio de Janeiro (Brazil), (22°58′12″S/43°14′30″ W, elevation of 452 m). The botanical identifications were carried out by Dr Elsie Franklin Guimarães of the Rio de Janeiro Botanical Garden Research Institute (JBRJ) and the voucher samples were deposited at the Herbarium RB/JBRJ under the identification code of RB01319727 for *P. gaudichaudianum* and RB01319723 for *P. mollicomum*. For establishing the monoclonal plantations, cuttings were collected in February 2017. For obtaining EOs, leaves were harvested from the wild population and the plantations in January 2018.

### 4.2. Species Propagation Protocol

Wild cuttings were collected from orthotropic branches of standard species, in three (n = 3) nodes, which were grown in a greenhouse at the Center for Social and Environmental Responsibility of the Botanical Garden of Rio de Janeiro, in the city of Rio de Janeiro, State of Rio de Janeiro (Brazil) in February 2017. Cuttings were disinfected with sodium hypochlorite at a concentration of 0.5% for 5 min, then washed with water, and finally dried with paper to remove excess moisture. The proximal ends of the cuttings were inserted 5 cm deep in 1 L plastic tubes, with the commercial substrate Tropstrato HT HORTALIÇAS^®^ for a period of 60 days.

The material was maintained with automatic irrigation and shading of 70%, until the formation of the root system, enabling transplantation. After rooting, the cuttings were transplanted into 10 L pots. When reaching 0.5 m, new cuttings were removed for planting the second generation, repeating the same procedure. The design was randomized, with twelve replications per species, and in triplicate. In January 2018, the two species and their surviving generations in cultivation reached length of approximately 90 cm. At this time, leaves were harvested to produce EOs.

Soil samples were obtained from Tijuca National Park where the two species were collected, considering five points near the base of each specimen, as described by Arruda et al., [84]. Chemical analysis of the soil, as well as of the commercial substrate Tropstrato HT HORTALIÇAS^®^ used in cultivation, was carried out by the Brazilian Agricultural Research Corporation-Soil Division (EMBRAPA-SOILS) (Table 1) according to Teixeira et al., [85].

### 4.3. Essential Oil Production

Leaves (100 g) were obtained from wild and cultivated species (1st and 2nd generation) of *P. mollicomum* and *P. gaudichaudianum*. Hydrodistillation was achieved in a Clevenger-type apparatus [86]. Fresh leaves were comminuted manually, with the aid of scissors, and placed in a 2L glass round bottom flask containing 700 mL distilled water. The flask was subjected to heating until boiling, and the procedure was continuous for 2 h [24,26]. After completion of the process, the pure EOs were separated from the aqueous phase, dried with anhydrous sodium sulfate, and stored in closed dark amber bottles in a freezer at −20 ºC until the time of analysis. Yields were calculated by the ratio of the volume in mL of oil and the weight in g of the fresh plant material used in the extraction, multiplied by 100, to express in percentage content [24,26]. The experiment was carried out in triplicate.

### 4.4. Essential Oil Analysis

Chemical characterization and quantification of the EOs of *P. mollicomum* and *P. gaudichaudianum* were carried out by Gas Chromatography (GC) coupled to Mass Spectrometry (MS) and GC coupled to the Flame Ionization Detector (GC-FID), respectively [24,26]. The EOs were diluted in dichloromethane (HPLC grade, TEDIA, Rio de Janeiro, Brazil) before analysis (1 mg/mL–1000 ppm). A sample of 1 μL of this solution was injected direct (splitless) into an HP AGILENT GC 6890 coupled to selective mass detector from the AGILENT MS 5973-N series, in which the injector temperature was set at 270 °C. Mass spectral ionization energy was set at 70 eV. An HP-5MS capillary column [AGILENT J & W; GC columns (Santa Clara, CA, USA)] with 30 m × 0.25 mm i.d. × 0.25 μm particle size was used. The chromatography conditions were 60 to 240 °C at 3 °C/min, totalling 60 min. Helium (~99.99%) was used as carrier gas at 1.0 mL/min in constant flow rate, and mass spectrometer operated at mass range from *m/z* 40 to 600 atomic mass units (*u*) [24,26].

GC-FID was obtained using a solution of 1 μL EO in dichloromethane (HPLC grade, TEDIA, Brazil, 1 mg/mL–1000 ppm), which was injected under the same analytical conditions described for GC-MS, except for the carrier gas used (hydrogen) with a constant flow rate of 1.0 mL/min. The retention times (Rt) of the compounds were measured in min, without correction, and used to calculate the linear retention indices (RI), that were obtained from the injection of a homologous series of *n*-alkanes (C_8_-C_25_; Sigma-Aldrich, Brazil), under the same analytical condition of the sample. Relative percentage areas were obtained from the GC-FID analysis [24,26,87]. Quantification was accomplished using sensitivity values determined from calibration curves that were developed with the use of external standards [24,26].

For compound identification, the mass spectral fragmentation pattern of the constituents was compared to the commercial GC-MS library NIST 98 and WILEY 7*n* [24,26] and the RI values were compared with those published in the literature [88]. In addition, co-injection with authentic standard was carried out wherever possible as described previously [21].

### 4.5. Evaluation of Chemodiversity and Micromolecular Parameters

To evaluate the chemical diversity of the EOs, to facilitate comparison between wild and intergenerational cultivated species, the Shannon index was used [89], as follows:

$$H^{\prime }=-\sum P_{i}{\mathrm{lnP}}_{i}$$

Although Shannon’s index is normally used to determine species diversity in an ecosystem, by treating each compound identity as a species and its relative abundance as the total number of species, chemical diversity could be calculated. In these equations, P_i_ is equivalent to the proportional abundance of the respective compound, which is obtained by dividing the quantity as determined by GC-FID by the total number of compounds identified in the sample, of which i is that number.

To characterize the micromolecular oxidation-reduction of the EO mixture, Ramos and Moreira’s index for mixtures was applied [21]. The equation is given below:

$${\mathrm{GM}}_{\mathrm{OR}}=\frac{\sum N_{\mathrm{OR}}}{N_{\mathrm{IA}}}$$

In these equations, the N_OR_ is the weighted oxidation state of the substance of interest and it is obtained by multiplying by the quantitative value of the substance found in the sample and divided by the number of carbon atoms in the molecular skeleton (n). The GM_OR_ is then obtained by the sum of N_OR_ of all substances in the mixture, divided by the number of identified substances (N_IA_) in the sample. A lower GM_OR_ indicates that the mixture has a lower average oxidation state by comparison with a sample that has a higher GM_OR_ [21].

### 4.6. Statistical Analysis

All data on the percentage of compounds in the EO were reported as mean ± standard deviation for three independent experiments (extraction). For the qualitative statistical analysis, the data referring to the constituents of the EO were submitted to the *Venn* diagram to verify the degree of similarity between the samples. Statistical significance was assessed using the Tukey test (ANOVA by Tukey HSD post hoc test). The chemometric analysis, principal component analysis (PCA) and hierarchical analysis (HCA) were used to assess the variance between the EO of wild and intergenerational cultivated species. The results were processed using STATISTICA software version 10 (StartSoft Inc., Tulsa, OK, USA).

## 5. Conclusions

The cultivation of *P. mollicomum* drastically changed its volatile composition. Therefore, before cultivating *P. mollicomum* it is necessary to identify the chemotype of the specimen and establish a study to determine the growing conditions and the factors that influence the essential oil production. *P. gaudichaudianum* showed almost the same volatile compounds in wild and cultivated specimens, which suggests that this plant tends to be less externally influenced. Our findings shed some light on issues that matter in the management, use and conservation of these two species of *Piper*, widely used as medicinal applications, in addition to contributing to the ecological and chemophenetic knowledge of these Piperaceae.

## Figures and Tables

**Table 1:** Results of the analysis of essential oils from *Piper mollicomum* (PM), referred to as wild, first- and second-generation cultivars (PMW, PMF and PMS, respectively), and *Piper gaudichaudianum* (PG) referred to as wild, first- and second-generation cultivars (PGW, PGF and PGS, respectively).

No. ^a^	Compounds ^b^	RI_lit_	RI_calc_	Relative Concentration (%) ± Standard Deviation ^c^					
				PGW	PGF	PGS	PMW	PMF	PMS
1	(3*H*)-Hexanol	844	844	0.03 ± 0.01					
2	**α-Pinene** ^#^	932	928	0.02 ± 0.02			**15.20 ± 0.03**	1.07 ± 0.02	
3	Camphene	946	954	0.04 ± 0.00					
4	**β-Pinene** ^#^	974	979		0.31 ± 0.02		**12.10 ± 1.03**	0.68 ± 0.08	
5	α-Phellendrene	1002	1000				1.19 ± 0.09	0.62 ± 0.04	
6	**1,8-Cineole** ^#^	1026	1024				**34.1 ± 1.54**	0.83 ± 0.05	
7	Limonene	1024	1026		0.12 ± 0.03	0.03 ± 0.02	2.14 ± 0.12		
8	*Z*-β-Ocimene	1032	1035				0.19 ± 0.02		
9	*E*-β-Ocimene	1044	1048				0.09 ± 0.03		
10	*Z*-Linalool oxide	1067	1069				0.16 ± 0.02	**18.95 ± 0.74**	0.34 ± 0.02
11	*E*-Linalool oxide	1084	1083					1.92 ± 0.08	
12	α-Terpinolene	1086	1089				0.30 ± 0.03		
13	**Linalool** ^#^	1095	1094	0.02 ± 0.01			**7.26 ± 0.46**	**37.88 ± 1.01**	**36.99 ± 1.32**
14	*E*-Pinocarveol	1135	1138				0.11 ± 0.03		
15	Camphor	1141	1143		1.23 ± 0.02	0.31 ± 0.03	0.10 ± 0.01		
16	Camphene hydrate	1145	1152				0.16 ± 0.02		
17	Pinocarvone	1160	1162				0.79 ± 0.03		
18	δ-Terpineol	1162	1164				4.69 ± 0.02		
19	Borneol	1165	1170	0.12 ± 0.03					
20	Terpinen-4-ol	1174	1174				0.87 ± 0.03		
21	α-Terpineol ^#^	1186	1190	0.23 ± 0.02	**4.87 ± 0.02**	**5.62 ± 0.02**	0.07 ± 0.02		
22	1-Tridecene	1290	1291				0.14 ± 0.03		
23	2-Undecanone	1293	1294				0.06 ± 0.02	0.31 ± 0.03	0.32 ± 0.02
24	δ-Elemene	1335	1337	2.31 ± 0.03	1.34 ± 0.04	1.87 ± 0.33	1.07 ± 0.01	0.99 ± 0.06	
25	Benzyl butanoate	1343	1345						1.08 ± 0.03
26	α-Cubebene	1345	1352	0.32 ± 0.02	0.98 ± 0.06	1.89 ± 0.02	0.09 ± 0.01		
27	α-Ylangene	1373	1374		0.78 ± 0.05		0.10 ± 0.02		
28	α-Copaene	1374	1376	1.23 ± 0.05	1.45 ± 0.03	1.67 ± 0.07	0.17 ± 0.01		
29	β-Bourbonene	1387	1383		0.32 ± 0.02	0.89 ± 0.08	1.80 ± 0.34		
30	β-Elemene	1389	1388	1.23 ± 0.04	1.37 ± 0.03	1.67 ± 0.04	2.13 ± 0.03	1.61 ± 0.05	1.15 ± 0.13
31	α-Gurjunene	1409	1409	1.45 ± 0.06	0.32 ± 0.07				
**32**	***E*-Caryophyllene** ^#^	1417	1418	**2.34 ± 0.04**	**5.43 ± 0.06**	**8.43 ± 0.07**		2.44 ± 0.22	2.49 ± 0.55
33	β-Gurjunene	1431	1435	0.45 ± 0.02	0.23 ± 0.02	0.21 ± 0.03	1.47 ± 0.22		
34	γ-Elemene	1434	1438	0.78 ± 0.06	0.03 ± 0.01		0.08 ± 0.03		
35	α-Guaiene	1437	1439					0.40 ± 0.06	
36	Aromadendrene	1439	1441	1.23 ± 0.07	1.04 ± 0.03	1.65 ± 0.76	0.17 ± 0.03		0.79 ± 0.02
37	*Z*-β-Farnesene	1440	1442		0.57 ± 0.02	3.43 ± 0.06			
38	*Z*-Muurola-3,5-diene	1448	1450				0.24 ± 0.03	0.89 ± 0.06	
39	α-Humulene	1452	1453	1.21 ± 0.06	0.32 ± 0.02		1.22 ± 0.03	2.22 ± 0.12	2.47 ± 0.04
40	*E*-β-Farnesene	1454	1454			1.73 ± 0.08			
41	*E*-Muurola-3,5-diene	1454	1455					0.21 ± 0.05	
42	β-Santalene	1457	1459				0.34 ± 0.01		
43	*Allo*-Aromadendrene	1458	1461	2.34 ± 0.04			0.07 ± 0.01	0.50 ± 0.04	0.54 ± 0.02
44	9-*epi*-*E*-Caryophyllene	1464	1468		1.23 ± 0.03	0.43 ± 0.02	0.33 ± 0.02		
45	γ-Muurolene	1478	1477	0.23 ± 0.00		1.21 ± 0.00			
46	Amorpha-4,7(11)-diene	1479	1478		0.23 ± 0.02				
47	*Ar*-Curcumene	1479	1480	0.08 ± 0.02			0.14 ± 0.01	0.18 ± 0.02	0.37 ± 0.03
48	α-Amorphene	1483	1483	5.21 ± 0.04	1.43 ± 0.34	0.76 ± 0.04	0.20 ± 0.01		
49	Germacrene D	1484	1484	0.05 ± 0.01	0.32 ± 0.03		0.57 ± 0.04	0.60 ± 0.09	
50	*Z*-Eudesma-6,11-diene	1489	1489	**4.32 ± 0.35**	**2.31 ± 0.12**	**3.34 ± 0.31**			
51	β-Selinene	1489	1490	3.45 ± 0.06	1.23 ± 0.03	1.87 ± 0.02	0.14 ± 0.03	0.72 ± 0.04	0.54 ± 0.01
52	δ-Selinene	1492	1492				0.14 ± 0.04		0.52 ± 0.05
53	*E*-Muurola-4(14),5-diene	1493	1493						
54	γ-Amorphene	1495	1496	4.21 ± 0.00					
55	α-Selinene	1498	1497	4.87 ± 0.00	3.25 ± 0.00	1.01 ± 0.00			
56	2-Tridecanone	1495	1497						0.29 ± 0.02
57	**Bicyclogermacrene** ^#^	1500	1499	**14.23 ± 0.0**	**16.12 ± 0.00**	**28.16 ± 0.00**	0.81 ± 0.06	1.34 ± 0.05	1.30 ± 0.03
58	α-Muurolene	1500	1501	0.76 ± 0.00	1.23 ± 0.00		0.06 ± 0.01		
59	*E*-β-Guaiene	1502	1503				0.20 ± 0.02		
60	*E*,*E*-α-Farnesene	1505	1505				0.49 ± 0.06	0.43 ± 0.02	
61	Cubebol	1515	1514					0.18 ± 0.06	0.38 ± 0.02
62	γ-Cadinene	1513	1515	1.23 ± 0.11	2.32 ± 0.27		0.52 ± 0.02	0.23 ± 0.01	0.39 ± 0.01
63	7-*epi-*α-Selinene	1520	1521	1.23 ± 0.05	1.23 ± 0.04				
64	δ-Cadinene	1522	1522	5.67 ± 0.08	3.56 ± 0.05		0.18 ± 0.03	0.81 ± 0.08	1.36 ± 0.07
65	*Z*-Calamenene	1528	1527						0.21 ± 0.03
66	Zonarene	1528	1530						0.24 ± 0.04
67	*E*-γ-Bisabolene	1529	1532	0.03 ± 0.02					
68	*E*-Cadina-1,4-diene	1533	1534	1.87 ± 0.08	0.67 ± 0.02	0.31 ± 0.04			
69	α-Cadinene	1537	1537	1.98 ± 0.06	0.89 ± 0.04				
70	Selina-3,7(11)-diene	1545	1546	1.45 ± 0.08	1.94 ± 0.09	2.31 ± 0.06	0.18 ± 0.02		
71	Elemol	1548	1551				0.08 ± 0.02	0.38 ± 0.10	
72	Germacrene B	1559	1558		1.23 ± 0.04	2.23 ± 0.05	0.12 ± 0.02		
73	*E*-Nerolidol	1561	1563	**12.11 ± 1.0**	**6.32 ± 0.21**	**8.03 ± 0.09**	2.12 ± 0.06	2.14 ± 0.09	**11.39 ± 1.04**
74	β-Calacorene	1564	1566		0.09 ± 0.02				
75	Palustrol	1567	1568	0.09 ± 0.02				0.22 ± 0.03	0.31 ± 0.02
76	Spathulenol	1577	1573		5.32 ± 0.02	3.32 ± 0.02		0.82 ± 0.04	1.13 ± 0.04
77	Caryophyllene oxide	1582	1581	1.23 ± 0.02				1.14 ± 0.12	2.07 ± 0.02
78	Globulol	1590	1585				0.50 ± 0.02		1.94 ± 0.01
79	Gleenol	1586	1586				0.36 ± 0.02		
80	Viridiflorol	1592	1594	**5.43 ± 0.05**	**7.89 ± 0.56**	**5.21 ± 0.40**	0.10 ± 0.01	0.57 ± 0.05	1.17 ± 0.07
81	Guaiol	1600	1602						0.35 ± 0.04
82	Ledol	1602	1604	5.08 ± 0.02	5.54 ± 0.07				
83	5-*epi*-7-*epi*-α-Eudesmol	1607	1607		0.32 ± 0.01				
84	Humulene epoxide II	1608	1607						2.08 ± 0.03
85	2,(7*Z*) -Bisaboladien-4-ol	1618	1618					0.21 ± 0.03	
86	10-*epi*-γ-Eudesmol	1622	1622						0.39 ± 0.02
87	*E*-Isolongifolanone	1625	1625						0.18 ± 0.03
88	1-*epi*-Cubenol	1627	1629				0.06 ± 0.02	0.48 ± 0.02	1.20 ± 0.01
89	*E*-Sesquilavandulol	1631	1630						1.11 ± 0.03
90	γ-Eudesmol	1630	1631				0.28 ± 0.02	1.68 ± 0.16	
91	Eremoligenol	1629	1632	0.04 ± 0.03					
92	*epi*-α-Cadinol	1638	1636						3.65 ± 0.19
93	*Z*-Cadin-4-en-7-ol	1635	1637					0.62 ± 0.08	
94	Caryophylla-4(12),8(13)-dien-5α-ol	1639	1637					0.32 ± 0.02	0.50 ± 0.08
95	*epi*-α-Muurolol	1640	1641					1.99 ± 0.09	1.03 ± 0.09
96	α -Muurolol	1644	1645	3.42 ± 0.04	0.98 ± 0.00		0.28 ± 0.03	0.60 ± 0.19	3.66 ± 0.02
97	α-Eudesmol	1652	1652	1.21 ± 0.05	0.56 ± 0.04				
98	α-Cadinol	1652	1653	**3.21 ± 0.12**	**9.32 ± 1.12**	**7.32 ± 0.68**	0.20 ± 0.01	2.22 ± 0.07	
99	*neo*-Intermedeol	1658	1658				0.08 ± 0.01		
100	Selin-11-en-4-α-ol	1658	1660				0.26 ± 0.03		
101	7-*epi*-α-Eudesmol	1662	1663		0.32 ± 0.02		0.09 ± 0.01		0.34 ± 0.04
102	Intermedeol	1665	1667		1.34 ± 0.01	2.32 ± 0.03	0.22 ± 0.03		
103	14-hydroxy-9-*epi*-*E*-Caryophyllene	1668	1672				0.63 ± 0.01	1.14 ± 0.08	
104	β-Bisabolol	1674	1677				0.24 ± 0.03		
105	α-Bisabolol	1685	1684				0.07 ± 0.01		
106	Eudesm-7(11)-en-4-ol	1700	1699					0.76 ± 0.06	
107	Benzyl benzoate ^#^	1759	1762				0.11 ± 0.01	1.05 ± 0.09	**5.69 ± 0.09**
**Monoterpene hydrocarbons**				0.06	0.43	0.03	31.10	2.39	0.00
**Oxygenated monoterpenes**				0.37	6.10	5.93	48.30	59.55	37.33
**Sesquiterpene hydrocarbons**				65.73	53.46	65.07	13.00	13.17	12.37
**Oxygenated sesquiterpenes**				31.81	37.91	26.20	5.57	15.45	32.88
**Other compounds**				0.03	0.00	0.00	0.31	1.36	7.38
**Identified Compounds in Numbers**				43	44	28	63	40	37
**Total compounds (%)**				98.01	97.90	97.23	98.34	92.32	89.96
**Oil yielding (%)**				0.17 ± 0.03	0.13 ± 0.02	0.18 ± 0.02	0.86 ± 0.08	0.12 ± 0.03	0.17 ± 0.06
**Shannon index**				3.81	3.78	3.30	4.14	3.86	3.67
**‘Ramos and Moreira Index (GM_RO_)**				−3.39	−3.46	−6.94	−2.55	−3.49	−3.73

RIcalc = Calculated Retention Index (HP-5MS column); RIlit = Literature Retention index–Adams^36^ was utilized; Main constituents in bold. SD= Standard Deviation. ^a^ Elution order on HP-5MS column; ^b^ All compounds were identified by MS and RI in accordance with experimental verses published values. ^c^ Quantities are averaged out of three replicates. Data are given as the mean ± standard deviation of three replicates. ^#^ Identification was by mass spectra, GC retention indices, comparison with literature data and co-injection with authentic compounds.

**Table 2:** Soil analysis results for *Piper mollicumum* (PM) and *Piper gaudichaudianum* (PG) in the Tijuca Forest/RJ and of the commercial substrate Tropstrato HT Hortaliças^®^ (THT).

Soil Attributes	*PM*	*PG*	THT
pH in Water	4.90 ± 0.95	5.40 ± 0.09	5.80 ± 0.18
Total acidity (cmolc/dm^3^)	11.88 ± 2,63	12.15 ± 0.89	8.91 ± 0.24
Al (cmolc/dm^3^)	0.10 ± 0.05	0.00 ± 0.00	0.00 ± 0.00
Ca (cmolc/dm^3^)	2.30 ± 0.09	2.00 ± 0.18	14.40 ± 5.89
Mg (cmolc/dm^3^)	1.30 ± 0.09	1.60 ± 0.08	6.90 ± 0.08
Na (mg/dm^3^)	18.40 ± 0.12	11.50 ± 1.70	27.60 ± 3.75
K (mg/dm^3^)	276.90 ± 32.45	202.90 ± 25.12	557.70 ± 41.04
P (mg/dm^3^)	7.54 ± 1.09	6.31 ± 1.07	25.33 ± 3.05
C (g/kg)	44.00 ± 6.31	67.00 ± 9.78	107.50 ± 13.43
N (g/kg)	4.30 ± 0.19	3.80 ± 0.13	4.20 ± 0.29
Cu (mg/dm^3^)	2.04 ± 0.04	2.77 ± 0.06	0.26 ± 0.08
Fe (mg/dm^3^)	29.70 ± 9.21	49.50 ± 8.45	23.60 ± 3.23
Mn (mg/dm^3^)	83.50 ± 4.32	82.40 ± 3.07	13.70 ± 1.45
Zn (mg/dm^3^)	4.43 ± 0.03	3.76 ± 0.04	3.46 ± 0.05
Value S (cmolc/dm^3^)	8.05 ± 0.032	7.15 ± 0.06	4.15 ± 0.32
Value T (cmolc/dm^3^)	19.93 ± 0.98	16.30 ± 0.21	6.30 ± 0.78
Value V (%)	40.39 ± 6.03	46.31 ± 4.20	65.93 ± 2.07

## Data Availability

Not applicable.
